# Differential toxic effects of bile acid mixtures in isolated mitochondria and physiologically relevant HepaRG cells

**DOI:** 10.1016/j.tiv.2019.104595

**Published:** 2019-12

**Authors:** Sophie L. Penman, Parveen Sharma, Hélène Aerts, B. Kevin Park, Richard J. Weaver, Amy E. Chadwick

**Affiliations:** a MRC Centre for Drug Safety Science, Department of Molecular and Clinical Pharmacology, University of Liverpool, Liverpool L69 3GE, UK; b Biologie Servier, 905 Rue de Saran, 45520 Gidy, France; c Institute de Recherches Internationales Servier, Biopharmacy, rue Carnot, 92284 Suresnes, France

**Keywords:** Bile acids, Drug-induced cholestasis, Mitochondria, HepaRG, Biliary transporters, ALR, ATP-linked respiration, BAs, Bile acids, BCA, bicinchoninic Acid, BR, basal respiration, BSEP, bile salt export pump, DIC, Drug-induced cholestasis, DILI, Drug-induced liver injury, DMEM, Dulbecco Modified Eagle Medium, FBS, Foetal bovine serum, FCCP, carbonyl cyanide 4-(trifluoromethoxy)phenylhydrazone, LDH, lactate dehydrogenase, MMP, mitochondrial membrane potential, MPT, mitochondrial permeability transition, MRC, maximum respiratory capacity, MRP, multi-drug resistance protein, NMR, non-mitochondrial respiration, OCR, Oxygen consumption rate, OXPHOS, oxidative phosphorylation, PBS, phosphate buffered saline, PCC, Pump controlled cell rupture, Pgp, P-glycoprotein, PL, proton leak, SEM, standard error of the mean, SRC, spare respiratory capacity

## Abstract

Bile acids (BAs) are recognised as the causative agents of toxicity in drug-induced cholestasis (DIC). Research in isolated mitochondria and HepG2 cells have demonstrated BA-mediated mitochondrial dysfunction as a key mechanism of toxicity in DIC. However, HepG2 cells are of limited suitability for DIC studies as they do not express the necessary physiological characteristics. In this study, the mitotoxic potentials of BA mixtures were assessed in isolated mitochondria and a better-suited hepatic model, HepaRG cells. BAs induced structural alterations and a loss of mitochondrial membrane potential (MMP) in isolated mitochondria however, this toxicity did not translate to HepaRG cells. There were no changes in oxygen consumption rate, MMP or ATP levels in glucose and galactose media, indicating that there was no direct mitochondrial toxicity mediated via electron transport chain dysfunction in HepaRG cells. Assessment of key biliary transporters revealed that there was a time-dependent reduction in the expression and activity of multi-drug resistance protein 2 (MRP2), which was consistent with the induction of cytotoxicity in HepaRG cells. Overall, the findings from this study have demonstrated that mitochondrial dysfunction is not a mechanism of BA-induced toxicity in HepaRG cells.

## Highlights

• HepaRG cells are a better suited in vitro model for cholestatic studies than HepG2 cell.
• Bile acids cause mitochondrial toxicity in isolated mitochondria but not in HepaRG cells.
• Time-dependent alterations in biliary transporters are consistent with the cytotoxicity of bile acid mixtures.
• There are important mechanistic differences when bile acids interact at the organelle level versus the whole cell.

## 1 Introduction

Drug-induced liver injury (DILI) presents both a clinical and a drug developmental problem as >1000 drugs have been associated with hepatotoxicity (Nathwani and Kaplowitz, 2006). DILI can present with multiple pathophysiology however, patterns of cholestatic injury occur in 20–40% of reported cases of DILI (Sgro et al., 2002). During drug-induced cholestasis (DIC), biliary transporter constraints can lead to an impairment in the flow of bile from the liver to the duodenum, resulting in the retention and accumulation of toxic levels of bile acids (BAs) within hepatocytes (Woolbright and Jaeschke, 2015).

Bile aids in the digestion of lipids and lipid-soluble vitamins within the small intestine by micellar formation and acts as a signalling molecule (Xie et al., 2001; Thomas et al., 2008). Despite these critical roles, when present at elevated levels, BAs can induce hepatotoxicity (Attili et al., 1986). BA hydrophobicity is a determinant of toxicity and protection, with the more hydrophobic BAs causing greater levels of hepatocyte injury (Perez and Briz, 2009). Research has shown that drugs with cholestatic potential can cause preferential BA accumulation, with an enhancement in hydrophobic BA accumulation (Sharanek et al., 2017). Investigation of the mechanisms associated with BA toxicity have elucidated a pathway of damage in which reactive oxygen species (ROS) generation and subsequent destruction of lipid membranes, bile canaliculi dynamic alterations, endoplasmic reticulum stress and mitochondrial toxicity leads to apoptosis or necrosis (Perez and Briz, 2009; Sharanek et al., 2016). Several studies using isolated mitochondria, rodent hepatocytes and HepG2 cells have demonstrated BA-mediated mitochondrial toxicity as a key event in DIC (Palmeira and Rolo, 2004; Rolo et al., 2004; Schulz et al., 2013). Much of this research has been conducted using single BAs and consequently overlooked the effect that a combination of BAs would have on hepatocytes, which is potentially more physiologically relevant to cholestatic injury in humans (Woolbright and Jaeschke, 2015).

Current in vitro predictions of DIC remain poor (Vinken, 2018). The principal test for detecting DIC is limited to the compounds ability to inhibit bile salt export pump (BSEP) in membrane vesicles (Hendriks et al., 2016). Whilst valuable for some drugs, this technique is of limited use as it lacks physiological relevance. Recent advancements have revealed that alterations in bile canaliculi dynamics are an early predictive marker of DIC (Burbank et al., 2016). Whilst valuable, there is still a need for further mechanistic insight into the pathology of DIC that would require an in vitro model with enhanced physiology. HepaRG cells have been postulated to be an improved model choice for DIC studies (Hendriks et al., 2016; Susukida et al., 2016; Woolbright et al., 2016). HepaRG cells are a bipotent progenitor cell line, capable of differentiating into hepatocytes and primitive biliary-like cells. Following differentiation, HepaRG cells have stable functionality for four weeks, allowing the assessment of a compounds cholestatic risk upon long-term, repeated exposure to be discovered (Cerec et al., 2007; Guillouzo et al., 2007). HepaRG cells share improved resemblance with primary human hepatocytes (PHH) than HepG2 cells as they express functioning bile canaliculi and have enhanced levels of phase 1–3 enzymes, albeit not identical (Aninat et al., 2006; Sison-Young et al., 2015). Therefore, the presence of these physiological characteristics enforce the suitability of HepaRG for DIC studies.

Whilst the mechanisms of BA toxicity in isolated mitochondria have been delineated, there is a lack of research into how these effects translate to the whole cell. It was therefore the aim of this research to define the role of the mitochondria in the onset of DIC in isolated mitochondria and HepaRG cells. The mitochondrial toxicity induced by BA mixtures were evaluated in HepaRG cells by comparing ATP levels and cytotoxicity after an acute metabolic modification, allowing mitochondrial toxicants to be detected as they cannot be masked by glycolytic ATP production (Swiss and Will, 2011; Kamalian et al., 2015). Further analysis of mitochondrial dysfunction in HepaRG cells was examined by alterations in mitochondrial membrane potential (MMP), followed by changes in oxygen consumption rate (OCR) using a Seahorse respirometer.

## 2 Materials and methods

### 2.1 Materials

HepG2 cells were acquired from European Collection of Cell Cultures (ECACC, Salisbury, UK). HepaRG cells, basal media, growth and differentiation additives were purchased from Biopredic International (Saint Grégoire, France). Dulbecco's modified media (DMEM) high glucose, fetal bovine serum (FBS), Cell Tracker 5- chloromethylfluorescein diacetate (CMFDA), NUPAGE 4–12% gels, rat tail collagen I and phosphate buffered saline (PBS) were purchased from Life Technologies (Paisley, UK). Nitrocellulose membrane and enhanced chemiluminescence (ECL) were purchased from GE Healthcare (Buckinghamshire, UK). All Seahorse consumables were purchased from Agilent (Santa Clara, USA). High precision pump – pump 11 was purchased from Harvard apparatus (Massachusetts, USA). 5,5′,6,6′-tetrachloro-1,1′,3,3′-tetraethyl benzimidazol carbocyanine iodide (JC-1) was purchased from Abcam (Cambridge, UK). Cytotoxicity Detection Kit was purchased from Roche Diagnostics Ltd. (West Sussex, UK). Balch homogeniser was purchased from Isobiotech (Heidelberg, Germany). Williams' E Medium powder (with l-Glutamine, without glucose) was manufactured by United States Biological. All bile acids, bile salts, MK571, bosentan were purchased from Sigma Aldrich (Dorset UK).

### 2.2 Cell culture

HepG2 cells were cultured at 37 °C in 5% CO_2_ in DMEM high-glucose medium (glucose; 25 mM) supplemented with 10% FBS (*v*/v), sodium pyruvate (1 mM), l-glutamine (2 mM) and HEPES (1 mM). HepG2 cells were used up to passage 20.

Undifferentiated HepaRG cells were supplied by the manufacturers at passage 12 and cultured as directed. Cells were thawed and grown in a T75 flask at 37 °C in 5% CO_2_ in HepaRG base medium supplemented with HepaRG growth additive for 2 weeks, with twice-weekly media changes. Cells were collected by trypsinisation and seeded into the appropriate assay plates at seeding densities supplied by the manufacturers. Cells were grown in growth media for 2 weeks. Following this, cells were maintained in HepaRG base media supplemented with HepaRG differentiation additive for 2 weeks, with twice-weekly media changes to complete the differentiation process. The cells remained in differentiation medium for an additional 4 weeks, in which all experiments were conducted. HepaRG cells were used up to passage 20.

### 2.3 Bile acid treatment

A mixture of the 6 most abundant BAs found within human plasma at physiological levels was prepared as the 1 x BA mixture (Xiang et al., 2010) (Table 1). In order to generate a dose-response, the concentrations of the individual BAs were increased to create a 10, 100 and 1000 x BA mix. HepaRG cells were treated for 24 and 72 h or 1 and 2 weeks (BAs were replaced in fresh media twice a week). BA mixtures were acutely applied to isolated mitochondria and remained for the duration of the assay (45 min). All stock solutions were prepared in DMSO and the final solvent concentration was kept at 0.5% (*v*/v), with a vehicle control included in each experiment.

**Table 1:** Composition of the BA mixtures and the concentrations of the individual BAs found within each mixture. In order to create a dose-response, the concentrations of the individual BAs were increased to reach a supra-physiological 1000 x BA mix.

Bile acid	Concentration in 1 x BA (μM)	Concentration in 10 x BA (μM)	Concentration in 100 x BA (μM)	Concentration in 1000 x BA (μM)
Cholic acid	0.41	4.1	41	410
Chenodeoxycholic acid	0.64	6.4	64	640
Deoxycholic acid	0.48	4.8	48	480
Lithocholic acid	0.008	0.08	0.8	8
Ursodeoxycholic acid	0.14	1.4	14	140
Glycochenodeoxycholic acid	0.8	8	80	800
Sum	2.478	24.78	247.8	2478

### 2.4 Isolation of mitochondria from HepG2 cells

2 fully confluent HepG2 cell T175 flasks were washed in Ca^2+^ free PBS, trypsinised, re-suspended in 4 °C isolation buffer (300 mM sucrose, 5 mM TES and 200 μM EGTA) at 7 × 10^6^ cells/ml and stored on ice for 15 min.

HepG2 mitochondria were isolated as described by the Pump Controlled Cell Rupture System (PCC) (Schmitt et al., 2013). A 6 μm tungsten carbide ball was inserted into a Balch homogeniser. 1 ml of cell suspension was passed through the homogeniser four times at a flow rate of 1400 μl/min. The homogenates were centrifuged at 800*g* for 5 min to pellet the cell debris and nuclei. The supernatant was retained and centrifuged at 9000*g* for 10 min to pellet the mitochondria. The PCC method and centrifugation steps were both conducted at 4 °C.

The mass of isolated mitochondria preparation was quantified using a standard Bradford assay and re-suspended in isolation buffer at 2 μg/μl (Bradford, 1976).

### 2.5 Mitochondrial membrane potential and structural alterations analysis in isolated mitochondria from HepG2 cells

Dual monitoring of MMP and structural alterations were performed simultaneously in a black 96-well plate with a transparent base. Mitochondria (50 μg) were loaded into the plate alongside 500 nm Rhodamine123 (Rh123) and acute BA mix treatment, which remained for the duration of the assay. MMP was monitored by the Rhodamine quenching method at excitation 500 nm, emission 528 nm (Zamzami et al., 2000). Rh123 accumulates in the matrix of mitochondria where energisation results in quenching of Rh123 fluorescence. Loss of MMP results in fluorescence recovery (Baracca et al., 2003). Mitochondrial structural alterations were assessed photometrically by light scattering at 540 nm where a reduction in optical density was indicative of mitochondrial swelling (Schulz et al., 2013). Both MMP and structural changes were monitored for 45 min on a plate reader (Varioskan, Thermo Scientific). Carbonylcyanide-p-(trifluoromethoxy) phenyl-hydrazone (FCCP) (10 μM) was used as a positive control for depolarisation and calcium (400 μM) was used as a positive control for mitochondrial swelling.

### 2.6 Mitochondrial membrane potential analysis in HepaRG cells

Undifferentiated HepaRG cells were plated onto collagen coated (50 μg/ml rat tail collagen type II in 0.02 M acetic acid) glass coverslides in 12-well plates at 80,000 cells/well. Following differentiation, MMP was monitored in HepaRG cells using the dye JC-1. HepaRG cells were treated with the BA mixtures for 24 h before incubation with JC-1 (1 μM, 1 h in the dark). Following this, cells were washed in PBS and incubated with Hoechst (1:5000 in PBS for 10 min). FCCP (100 μM) was used as a positive control for MMP depolarisation. Cells were mounted with Prolong gold onto glass coverslides. Images were taken using a Zeiss Axio Observer microscope with Apotome using 40 x oil objective with the excitation wavelength of 488 nm for green and 545 nm for red. The ratio of JC-1 red aggregate to green monomer was calculated and a decrease was determined as a loss of MMP.

### 2.7 Inhibition of biliary transporter activity in HepaRG cells

Undifferentiated HepaRG cells were plated onto collagen coated glass coverslides in 12-well plates at 80,000 cells/well. HepaRG cells were incubated with CMFDA (5 μM) and the cell-permeable DNA stain Hoechst (1:5000) with or without MK571 (30 μM, Multidrug resistance protein (MRP) inhibitor) and bosentan (50 μM, BSEP inhibitor) in HBSS for 30 min at 37 °C. CMFDA passively diffuses across the cell membrane. Within the cell it is converted into the impermeable MRP2 and P-glycoprotein (Pgp) substrate, glutathione-methylfluorescein (GSMF) (Forster et al., 2008). Cells were washed with HBSS to eliminate excess CMFDA and then mounted with Pro-long gold onto glass microslides. Snap images with Apotome were taken using a Zeiss microscope using 40 x oil objective.

### 2.8 Western blotting for the detection of transporter expression in HepaRG cells

Undifferentiated HepaRG cells were plated in collagen coated 6-well plates at 200,000 cells/well. Following differentiation, HepaRG cells were treated for 24 h with BA mixtures and then lysed in Radio-Immunoprecipitation Assay (RIPA) buffer.

Western blotting was carried out according to standard protocols. Briefly, 20 μg of total protein lysate was subjected to SDS-PAGE electrophoresis and the gel transferred to a nitrocellulose membrane. Incubation and dilutions for the primary and secondary antibodies were dependent on the protein of interest (Table 2). Protein bands were visualised using an ECL system.

**Table 2:** Western blot incubation conditions for primary and secondary antibodies used to assess transporter expression in HepaRG cells.

Protein	Molecular weight (kDa)	Primary antibody (in 5% milk)	Secondary antibody (in 5% milk)
MRP2	174	1:40	Anti-mouse
BSEP	146	1:750	Anti-rabbit
NTCP	38	1:100	Anti-rabbit
GAPDH	37	1:5000	Anti-mouse

### 2.9 Acute metabolic modification assay in HepaRG cells

Undifferentiated HepaRG cells were plated in collagen coated 96-well cell culture plates at 9000 cells/well. Following differentiation, cells were treated as described below.

Serum-free base media was prepared from glucose-free Williams E powder dissolved in sterile distilled water and supplemented with sodium bicarbonate (3.7 mg/ml), insulin (5 μg/ml) and hydrocortisone (50 μm). To make glucose and galactose media, the base was supplemented with galactose (10 mM) or glucose (11 mM), which is the same concentration of glucose found within the HepaRG differentiation media as told by Biopredic.

#### 2.9.1 24 h BA mix treatment

HepaRG cells were washed twice in either serum-free glucose or galactose media before incubation in the respective media (50 μl, 2 h). BA mixtures were diluted in either serum-free glucose or galactose media to reach a final solvent concentration of 1% (*v*/v) and added to every well of the plate (50 μl, 24 h).

#### 2.9.2 72 h, 1 week and 2 week BA mix dosing

HepaRG cells were dosed with the BA mixtures in HepaRG culture media (72 h, 1 week and 2 weeks). Cells were washed twice with either serum-free glucose or galactose media and then incubated in the respective media (100 μl, 2 h).

#### 2.9.3 Inhibition of biliary transporters with MK571 and bosentan

HepaRG cells were washed twice with either serum-free glucose or galactose media before incubation in the respective media (50 μl, 2 h). Bosentan (50 μM) and MK571 (30 μM) were diluted in either serum-free glucose or galactose media to reach a final solvent concentration of 1% (v/v) and added to every well of the plate (50 μl, 30 mins). Following this, inhibitors were aspirated and HepaRG cells were treated in serum-free glucose or galactose media containing BA mixtures, bosentan and MK571 (100 μl, 24 h).

Following BA mix treatment (24 h) or the 2 h metabolic switch (72 h, 1 week and 2 week), the cell supernatant was removed and the cells lysed with somatic ATP releasing agent. ATP content and protein were measured as described previously (Kamalian et al., 2015; Kamalian et al., 2018).

### 2.10 Measuring cell toxicity over time using the lactate dehydrogenase assay in HepaRG cells

HepaRG cells were treated for 1 week with the BA mixtures. Following this, HepaRG cells were dosed with the BA mixtures daily for an additional week. The supernatant was collected and daily lactate dehydrogenase (LDH) content was measured using a Cytotoxicity Detection Kit in accordance with the manufacturer's instructions in order to determine the total LDH released into the supernatant. Following 2 weeks of BA mix dosing, the supernatant was removed and the cells were lysed in somatic ATP releasing agent. The lysate was diluted (1:5) using media and LDH content within the lysate was measured using a plate reader at 490 nm.

The LDH retained within the cells was determined by the following formula

$$\mathrm{Retained}\;\mathrm{LDH}=\mathrm{LDH}\;\mathrm{in}\;\mathrm{lysate}/\left(\mathrm{LDH}\;\mathrm{in}\;\mathrm{lysate}+\mathrm{LDH}\;\mathrm{in}\;\mathrm{supernatant}\right).$$

### 2.11 Seahorse XF mitochondrial stress test in HepaRG cells

Undifferentiated HepaRG cells were plated in collagen coated XF 96-well cell culture plates at 5000 cells/well. Following differentiation, HepaRG cells were dosed with BA mixtures.

A mitochondrial stress test was conducted as previously described in (Kamalian et al., 2018). Briefly, HepaRG cells were incubated with unbuffered Seahorse XF Base medium supplemented with glucose (25 mM), sodium pyruvate (1 mM) and l-glutamine (2 mM) adjusted to pH 7.4. Cells were incubated for 1 h in a non-CO_2_ incubator before the sequential injections of stress test compounds from ports A-C of the XFe96 sensor cartridge, oligoymcin (1 μM), FCCP (0.25 μM), rotenone (1 μM) and antimycin-A (1 μM).

The OCR results were normalised to protein content, calculated by a standard BCA assay (Smith et al., 1985). Normalised data was analysed using the Seahorse XF Mitochondrial Stress Test Generator from Agilent.

**Fig. 1:**
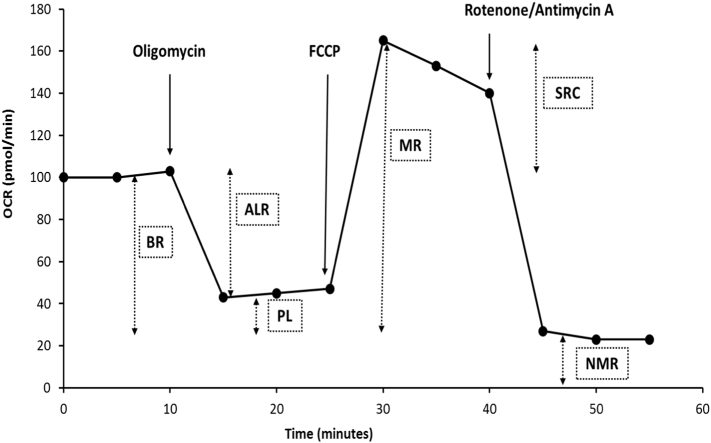
Illustration of a typical mitochondrial stress test only using injection ports A-C. Oligomycin is released from port A, FCCP from port B and rotenone and antimycin A from port C. OCR data can be manipulated to determine mitochondrial parameters such as basal respiration (BR), ATP-linked respiration (ALR), proton leak (PL), maximum respiration (MR), spare respiratory capacity (SCR) and non-mitochondrial respiration (NMR).

OCR values were used to calculate non-mitochondrial respiration (NMR = minimum rate measurement after rotenone/antimycin A injection), basal respiration (BR = last measurement before oligomycin injection – NMR), proton leak (PL = minimum measurement after oligomycin injection – NMR), ATP-linked respiration (ALR = BR – PL), maximum respiration (MR = maximum measurement after FCCP injection – NMR) and spare respiratory capacity (SRC = MR – BR) (Fig. 1).

### 2.12 Statistical analysis

Data is expressed from a minimum of three independent experiments. Normality was assessed using a Shapiro-Wilk statistical test. Statistical significance was determined by a one-way ANOVA with a Dunnett's test for parametric data or a Kruskal-Wallis test for non-parametric data using StatsDirect 3.0.171. Results were considered significant when *P <* *.*05.

## 3 Results and discussion

### 3.1 HepaRG cells are an appropriate model for DIC studies as they express functional biliary transporters

**Fig. 2:**
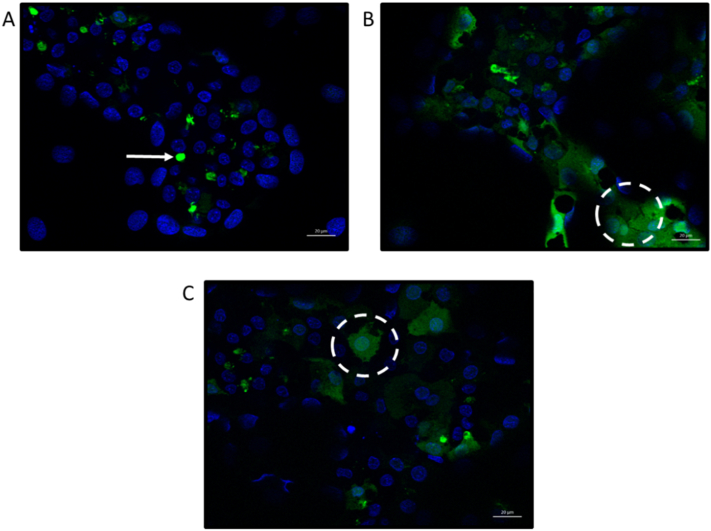
Transporter function and inhibition in HepaRG cells. HepaRG cells were incubated with (A) CMFDA (5 μM) and Hoechst (1:5000) only for 30 min; (B) CMFDA, Hoechst and the MRP inhibitor MK571 (30 μM) for 30 min; (C) CMFDA, Hoechst and the BSEP inhibitor bosentan (50 μM) for 30 min. Maximum intensity projection images with Apotome were taken using a Zeiss microscope using 40 x oil objective. Arrow indicates CMFDA within bile canaliculi whereas the circles indicate CMFDA retained within the cell cytoplasm. Scale bar = 20 μm.

The functionality of biliary transporters important in BA efflux were tested. HepaRG cells expressed functioning biliary transporters as seen by the efflux of CMFDA into the bile canaliculi by MRP2 and Pgp transporters (Fig. 2A). During DIC, biliary transporter constraints lead to a retainment of BAs within hepatocytes (Woolbright and Jaeschke, 2015). To test whether transporters could be inhibited, MRP and BSEP were blocked with known transporter inhibitors, MK571 and bosentan. Inhibition of MRP and BSEP prevented the efflux of CMFDA into the bile canaliculi and it was retained within the cell cytoplasm (Fig. 2B and C), indicating that HepaRG cells can be manipulated to recapture transporter inhibition as in DIC (Woolbright and Jaeschke, 2015). BA-mediated toxicity has been demonstrated using the hepatic line HepG2 cells (Perez and Briz, 2009). HepG2 cells have limited suitability for DIC studies as they do not express functional biliary transporters and many drugs with known cholestatic liabilities are inhibitors of BSEP and MRP transporters (Hofmann et al., 1999; Sison-Young et al., 2015). Furthermore, whilst some hydrophobic BAs can enter the cell via passive diffusion, active transporter-mediated uptake and efflux is also a major route of BA movement, thus requiring a model that can induce this movement (Hofmann et al., 1999).

### 3.2 High concentrations of BAs induce structural alterations and depolarisation in isolated mitochondria but not in HepaRG cells

During DIC, intracellular concentrations of BAs increase and are reported to cause toxicity. Research into BA toxicity in obstructive cholestasis found that there is potential leakage of bile back into the parenchyma that may expose hepatocytes to biliary concentrations of BAs, which are far greater than serum levels (Fickert et al., 2002; Woolbright et al., 2015). Whilst the concentrations of various BAs in serum rise to no >20 μM during cholestasis, biliary levels can reach up to 1–5 mM for certain BAs (Woolbright et al., 2015). In order to create a dose-response and observe how much deviation from healthy serum levels (1 x BA) was needed to induce toxicity, the supra-physiological 1000 x BA mix was used. Although the 1000 x BA mix is supra-physiological of serum levels, it is similar to the concentrations BAs are postulated to rise to in the bile, albeit not identical. Although it is suspected that biliary levels of BAs are responsible for hepatotoxicity in obstructive cholestasis, it remains unclear whether during DIC hepatocytes are exposed to such biliary fluids containing high levels of BAs. Previous research has shown that BAs can mediate mitochondrial toxicity in isolated mitochondria when tested individually (Krahenbuhl et al., 1994; Rolo et al., 2000; Schulz et al., 2013). Therefore, it was hypothesis that BA mixtures would lead to structural alterations and MMP depolarisation in isolated mitochondria.

**Fig. 3:**
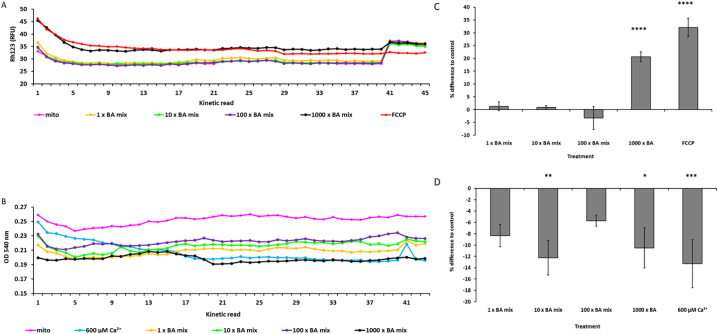
The effects of BA mixtures on MMP and optical density on isolated mitochondria from HepG2 cells. (A) Simultaneous measurements of MMP loss (Rh123 fluorescence) and (B) optical density (OD_540nm_) were conducted for 1 h. (C) Difference in MMP and (D) optical density at kinetic read 20 for mitochondria acutely treated with BA mixtures compared to control. FCCP served as an internal control for MMP loss and calcium served as a control for mitochondrial swelling. Statistical significance compared with control; * *P* < .05, ** *P* < .01, *** *P* < .001, ****P < .0001. Data are presented as ± SEM of *n* = 4 experiments.

Isolated mitochondria from HepG2 cells generated an inner transmembrane potential which remained stable for 1 h (Fig. 3A). Acute treatment with 1000 x BA mix resulted in a loss of MMP as indicated by a 20.7 ± 1.9% increase in fluorescence when compared to control mitochondria (Fig. 3C). At kinetic read number 40, FCCP was added to all wells to act as an internal control for depolarisation. This addition resulted in an increase in fluorescence for mitochondria treated with the 1, 10 and 100 x BA mixtures, indicating that initial BA mix treatment had not uncoupled the membrane potential (Fig. 3A).

Concomitant optical density measurements were used to assess whether BA mixtures altered the structure of the mitochondria. Calcium was included as a positive control for mitochondrial swelling, as high doses trigger the mitochondrial permeability transition (MPT) which is characterised by mitochondrial swelling leading to outer membrane rupture and cell death (Crompton, 1999; Gáll et al., 2012). All concentrations of the BA mixtures induced a decrease in optical density, implying mitochondrial swelling or structural alterations (Fig. 3B and D). However, BA mixtures did not result in induction of the MPT as mitochondria still had an intact membrane potential (Fig. 3A).

**Fig. 4:**
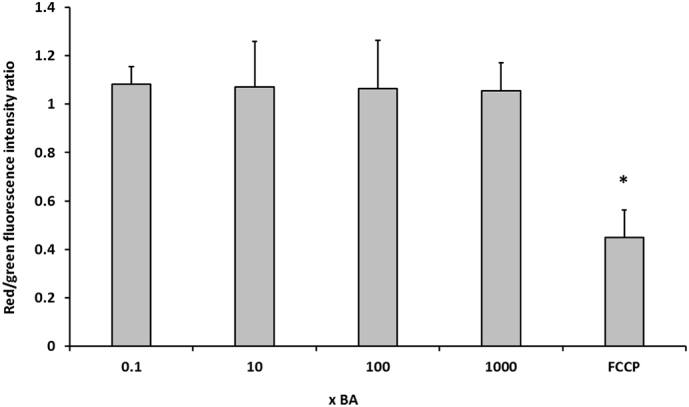
The effects of BA mixtures after 24 h on MMP. HepaRG cells were treated for 24 h with BA mixtures and MMP was detected by measurement of JC-1 fluorescence. A ratio of the fluorescence intensity of the red aggregate over the green monomer was determined. FCCP was used as a positive control for depolarisation. Statistical significance compared with vehicle control 1 week; PL * *P* < .05. Data are presented as + SEM of *n* = 3 experiments. (For interpretation of the references to colour in this figure legend, the reader is referred to the web version of this article.)

These results support previous findings in isolated mitochondria with single BAs, specifically that when administered in combination, BA mixtures caused mitochondrial toxicity (Rolo et al., 2000; Schulz et al., 2013). However, there were no differences in the red/green fluorescent ratio following 24 h BA mix treatment in HepaRG cells, suggesting that BA mixtures did not induce loss of MMP in whole cells (Fig. 4). The uncoupler FCCP was used as a positive control for MMP depolarisation. FCCP induced significant MMP loss with a decrease of 0.4 ± 0.1 in the red/green fluorescent ratio. Whilst BAs possess acid dissociable groups, the lack of depolarisation in HepaRG cells is not surprising as they lack other structural moieties present in FCCP that aid in its uncoupling activity. These include the presence of a bulky lipophilic group and strong electron withdrawing groups (Palmeira and Rolo, 2004). Therefore, the depolarisation seen in isolated mitochondria (Fig. 3C) could be attributed to a direct interaction and immediate access of the BAs on the mitochondria. Whereas in whole cells, processes such as conjugation and metabolism could produce less toxic entities that are chemically unable to induce depolarisation (Chiang, 2013).

### 3.3 BA mixtures exert time-dependent cytotoxicity that is not mediated via dysfunction of mitochondrial respiration in HepaRG cells

The mitochondrial dysfunction of BA mixtures were assessed using the modified glucose-galactose assay in HepaRG cells. The tumorigenic origin of many cell lines means that their energy demands can be met by both glycolysis and oxidative phosphorylation (OXPHOS). Whilst in high-glucose media, these cells use OXPHOS as their main source of ATP but if required can use glycolysis and consequently mask any mitochondrial dysfunction (Marroquin et al., 2007; Diaz-Ruiz et al., 2011). Substitution of glucose with galactose in the media causes a reduction in the yield of ATP generated from glycolysis and forces the cells to rely on OXPHOS for ATP generation, thus increasing susceptibility to mitochondrial toxicants (Marroquin et al., 2007; Kamalian et al., 2015). The aim of the glucose-galactose assay is to detect mitochondrial toxicity prior to the induction of cytotoxicity. ATP is measured at early time-points as a marker of mitochondrial function in the absence of cytotoxicity (Kamalian et al., 2015).

**Fig. 5:**
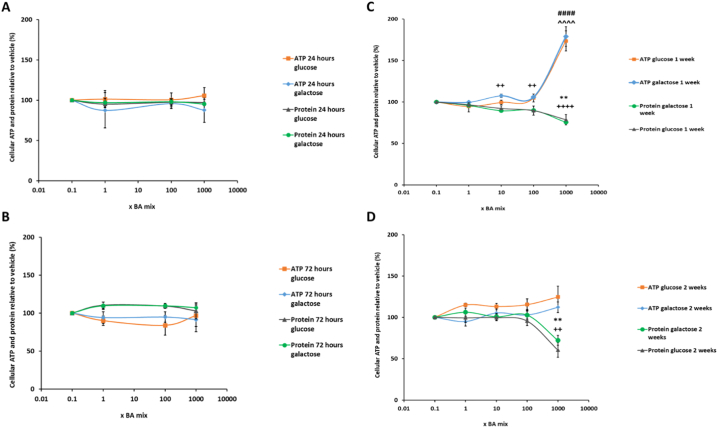
The effects of BA mix treatment after (A) 24 h, (B) 72 h, (C) 1 week and (D) 2 weeks on cellular ATP content and protein compared to the vehicle control. Statistical significance compared with vehicle control; ATP glucose ^ *P* < .05, ^^ *P* < .01, ^^^ *P* < .001, ^^^^ *P* < .0001, ATP galactose ^#^*P* < .05, ^##^*P* < .01, ^###^*P* < .001, ^####^*P* < .0001, protein glucose * *P* < .05, ** *P* < .01, *** *P* < .001, ****P < .0001 and protein galactose ^+^*P* < .05, ^++^*P* < .01, ^+++^*P* < .001, ^++++^*P* < .0001. ATP values have been normalised to μg protein per well. Data are presented as ± SEM of *n* = 4 experiments.

There were no significant differences in ATP levels between glucose or galactose media at any of the time points tested, thus suggesting that there was no direct mitochondrial toxicity mediated via electron transport chain dysfunction in HepaRG cells (Fig. 5). Using this technique it is stated that a compound can be defined as mitotoxic by calculating IC_50_ ATP values in glucose and galactose media. An IC_50_-ATPglu/IC_50_-ATPgal ≥2 is indicative of a compound with a mitochondrial liability (Swiss et al., 2013; Kamalian et al., 2015). IC_50_ values could not be determined for any of the time points as BA mix treatment did not result in a reduction in cellular ATP content, thus meaning that according to this assay BA mixtures are not mitotoxic (Fig. 5).

**Fig. 6:**
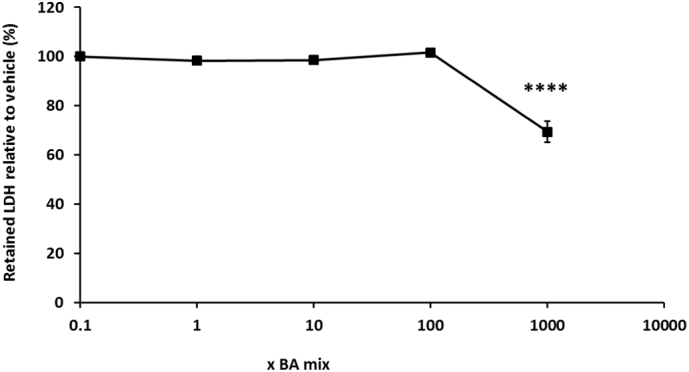
The effects of BA mixtures after 2 weeks treatment on cellular retained LDH levels compared to the vehicle control. Supernatant was collected daily after 1 week BA mix treatment and HepaRG cells re-dosed for an additional week. LDH content in the lysate was determined and retained LDH was calculated by; LDH in lysate/ (LDH in lysate + supernatant). Statistical significance compared with vehicle control; * *P* < .05, ** *P* < .01, *** *P* < .001, ****P < .0001. Data are presented as ± SEM of *n* = 3 experiments.

Protein was measured as a marker of cell death because once HepaRG cells have reached confluency and are fully differentiated, they do not continue proliferating and so any differences in protein can be attributed to cell death (Gripon et al., 2002). Protein gradually started to decrease after 1 week BA mix dosing in a dose-dependent fashion and after 2 weeks there was a significant loss of 39.5 ± 8.9% protein for the 1000 x BA mixture (Fig. 5D), suggesting a time-dependent cell death not mediated via mitochondrial dysfunction. Despite the loss of protein, ATP levels significantly increased up to 180.0 ± 11.9% (Fig. 5C) and then reduced to 124.6 ± 13.0% of the vehicle at 2 weeks BA treatment (Fig. 5D). HepaRG cells are a heterogeneous population containing both hepatocytes and primitive biliary-like cells (Marion et al., 2010). Images taken on the light microscope showed that there was a loss of the hepatocyte clusters in the HepaRG cells (supplementary data). This increase in ATP following 1 week of dosing could be attributed to the BAs killing the hepatocytes but the biliary-like cells being able to withstand cytotoxicity by switching off ATP consuming processes to maintain ATP stores for cellular defence mechanisms (Kamalian et al., 2015). LDH levels were assessed to gain further mechanistic understanding of the cause of toxicity. Daily collective supernatant values revealed that the 1000 x BA mixture induced significant cytotoxicity in HepaRG cells (Fig. 6). After 1 week BA dosing in glucose media there was a 30.6 ± 4.3% decrease in retained LDH, confirming that mitotoxicity does not precede cytotoxicity and that the BAs were causing toxicity via another mechanism.

**Fig. 7:**
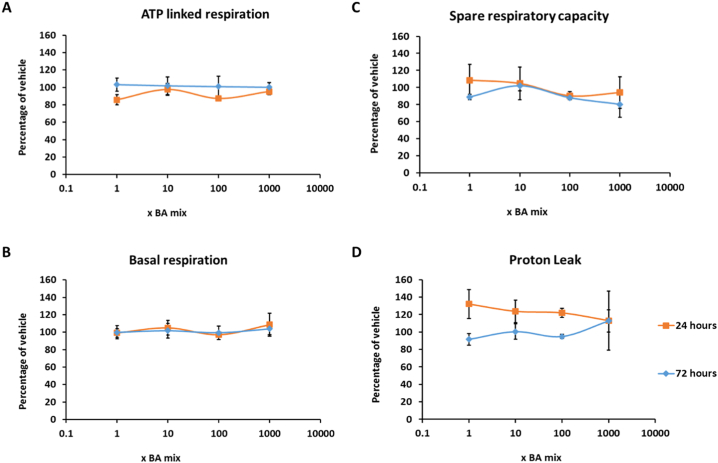
The effects of BA mixtures after 24 and 72 h treatment on mitochondrial OCR in HepaRG cells. Mitochondrial parameters; (A) ATP-linked respiration, (B) Basal respiration, (C) Spare respiratory capacity and (D) Proton leak, were calculated from OCR data to allow in-depth analysis of mitochondria after BA treatment. All results were normalised to μg of protein per well. Data are presented as ± SEM of *n* = 4 experiments.

**Fig. 8:**
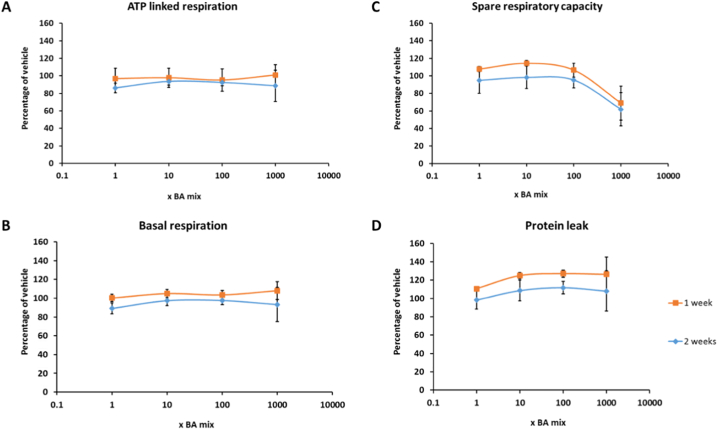
The effects of BA mixtures after 1 and 2 weeks treatment on mitochondrial OCR in HepaRG cells. BA mixtures were replenished twice weekly. Mitochondrial parameters; (A) ATP-linked respiration, (B) Basal respiration, (C) Spare respiratory capacity and (D) Proton leak, were calculated from OCR data to allow in-depth analysis of mitochondria after BA treatment. All results were normalised to μg of protein per well. Data are presented as ± SEM of n = 4 experiments.

The glucose-galactose assay is useful in detecting compounds that cause mitochondrial dysfunction of the electron transport chain however, it is important to note that it is restricted in its ability to detect compounds that cause mitochondrial dysfunction via alternative mechanisms such as reactive metabolite production or inhibition of fatty acid oxidation (Kamalian et al., 2015). Therefore, if a compound is deemed negative for mitotoxicity, further respiratory analysis should be undertaken for verification. Seahorse technology enables respiratory parameters to be measured in real time, thus allowing a more in-depth insight into the mechanisms of toxicity of compounds. BA mixtures did not cause a significant change in any of the parameters of mitochondrial function measured, suggesting that the BAs do not induce mitochondrial dysfunction (Fig. 7, Fig. 8). Previous analysis of mitotoxins by Seahorse respirometry have demonstrated that decreases in SRC are the most sensitive parameter and decreases often signal as a warning for subsequent mitochondrial dysfunction (Kamalian et al., 2018). Although there was a dose-dependent decrease in SRC in cells that had been dosed for 1 and 2 weeks (Fig. 8C), decreasing to 68.9 ± 19.2% for 1 week and 61.9 ± 18.8% for 2 weeks, this was not significant and may be a result of cell death as measured previously.

In order to confirm that the composition of the BAs was not responsible for the absence of mitochondrial toxicity, a second mixture composed of the BAs found within the bile of patients with cholestatic liver injury was prepared (Woolbright et al., 2015). The mitochondrial toxicity of this second BA mix was assessed in isolated mitochondria and HepaRG cells and emulated the data of the initial BA mixtures confirming that BAs do not cause mitochondrial dysfunction in HepaRG cells (Supplementary data).

### 3.4 Inhibition of biliary transporters in HepaRG cells does not lead to the establishment of mitochondrial toxicity by BA mixtures

HepaRG cells express functional biliary transporters (Le Vee et al., 2006). Therefore it was hypothesised that the absence of mitochondrial toxicity in HepaRG cells could be attributed to a lack of a static BA pool within the hepatocytes due to their continuous influx and efflux by the functional biliary system. As previously shown, MK571 and bosentan inhibit MRP2 and BSEP respectively in HepaRG cells and lead to a retainment of CMFDA within the cell cytoplasm (Fig. 2). In order to prevent BA efflux and create a static BA pool within the hepatocytes, HepaRG cells were pre-treated with MK571 and bosentan and then subsequently dosed with the BA mixtures for 24 h.

**Fig. 9:**
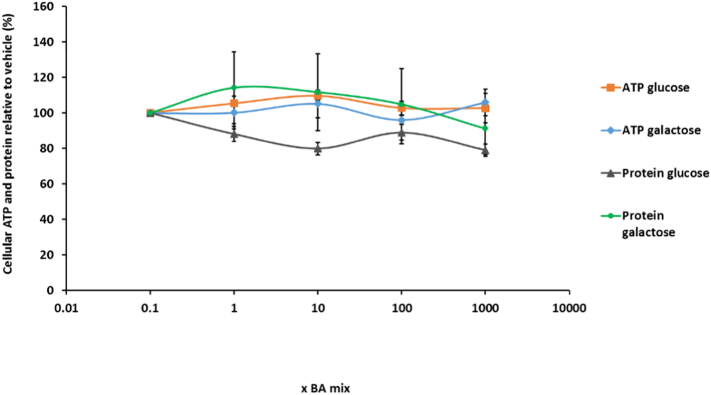
The effects of BA mixtures after 24 h treatment following biliary transporter inhibition by MK571 (MRP inhibitor) and bosentan (BSEP inhibitor) on ATP content and protein compared to the vehicle control. ATP values have been normalised to μg protein per well. Data are presented as ± SEM of *n* = 5 experiments.

**Fig. 10:**
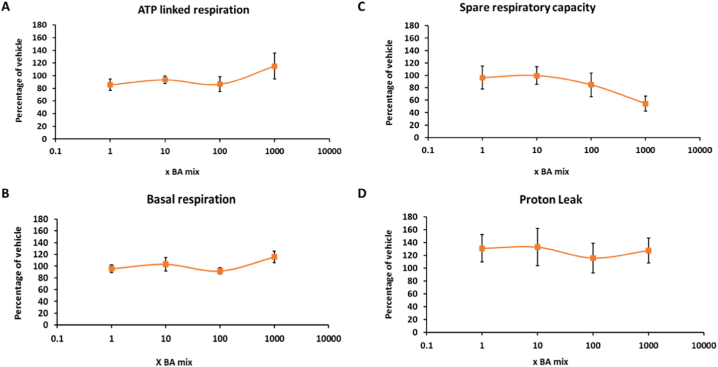
The effects of BA mixtures after 24 h treatment following biliary transporter inhibition by MK571 (MRP inhibitor) and bosentan (BSEP inhibitor) on mitochondrial OCR in HepaRG cells. Mitochondrial parameters; (A) ATP-linked respiration, (B) Basal respiration, (C) Spare respiratory capacity and (D) Proton leak, were calculated from OCR data to allow in-depth analysis of mitochondria after BA treatment. All results were normalised to μg of protein per well. Data are presented as ± SEM of *n* = 3 experiments.

In the presence of the transporter inhibitors, there were no significant changes in ATP content or protein levels in the acute metabolic modification assay (Fig. 9). Furthermore, respirometry results demonstrated that there were no significant changes to any of the parameters measured (Fig. 10), although again there was a non-significant dose-dependent decrease in SRC to 54.4 ± 12.0% following 1000 x BA mix treatment (Fig. 10C).

Overall, these results demonstrate that inhibition of biliary transporters in order to supply a pool of static BAs to the hepatocytes did not expose any mitochondrial liabilities of the BA mixtures. It is important to note that under physiological conditions BA efflux via the basolateral membrane is negligible, however during cholestasis the expression of MRP3 and MRP4 are upregulated to act as a compensatory mechanism of efflux (Trauner and Boyer, 2003). MK571 is an inhibitor of the MRP family of transporters and has been shown to inhibit MRP3 and MRP4, as well as MRP2 (Gekeler et al., 1995; Weiss et al., 2007; Lien et al., 2014; Tivnan et al., 2015). Therefore incubation of HepaRG cells with MK571 and bosentan would have prevented all major routes of BA efflux and led to the establishment of a pool of BAs within the cells. These results suggest that even when retained within hepatocytes, BA mixtures do not cause mitochondrial toxicity in HepaRG cells.

### 3.5 BA mixtures exert time-dependent changes in the function and expression of biliary transporters

**Fig. 11:**
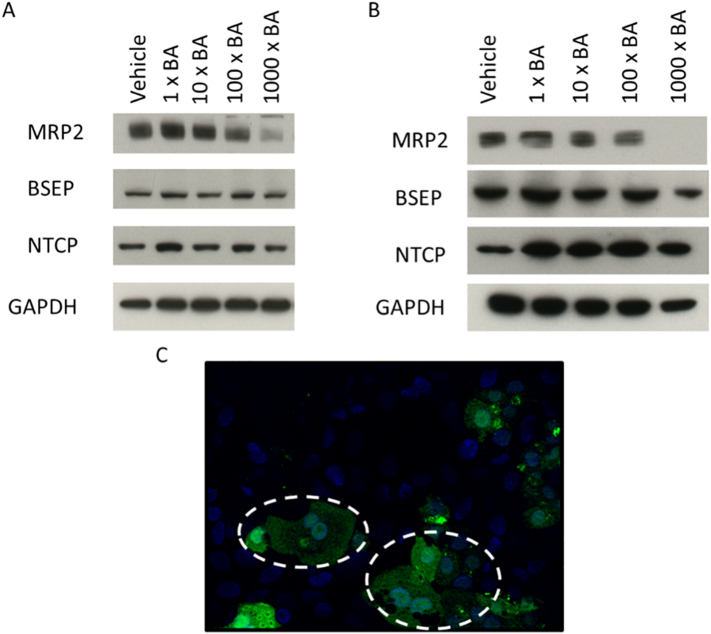
The temporal effects of BA mixtures on transporter activity and expression. (A) Transporter expression following 24 h BA mix treatment (B) Transporter expression following 2 weeks BA mix treatment and (C) CMFDA retainment within the cytoplasm (circled) due to failed activity of MRP2 and Pgp transporters following 24 h 1000 x BA mix treatment.

The expression of key transporters important in BA influx and efflux were measured over time following BA mix treatment. The results revealed that there was a time-dependent reduction in the expression of MRP2 with the 1000 x BA mix, whilst the expression of other transporters remained unchanged (Fig. 11A and B). The expression of MRP2 started to decrease following 24 h treatment and after 2 weeks there was negligible expression following 1000 x BA mix treatment (Fig. 11B). Additionally, after 24 h treatment, 1000 x BA mix led to a retainment of CMFDA in the cells indicating that the activity of MRP2 and Pgp had been reduced (Fig. 11C). It is also of note that a reduction in MRP2 occurs in patients with cholestasis (Kullak-Ublick et al., 2000; Chai et al., 2015). In vivo, a loss of MRP2 could lead to clinical implications such as conjugated hyperbilirubinemia and jaundice (Paulusma et al., 1996; Hashimoto et al., 2002; Kikuchi et al., 2002). In this experiment, the reduction in expression and activity of MRP2 was consistent with the onset of cytotoxicity. However, due to compensatory mechanisms of BA efflux, this reduction is unlikely attributable to the cytotoxicity seen in HepaRG cells (Trauner and Boyer, 2003; Keppler, 2014).

The results from this experiment show that BAs exert a temporal cytotoxicity that is not mediated via mitochondrial dysfunction. A recent publication in HepaRG cells found that bile salt treatment caused a decrease in the expression and release of albumin and a reduction in the biliary excretory capacity as seen in this study (Messner et al., 2019). Biomarker analysis revealed that levels of the hepatocyte-specific marker miR-122 were elevated thus suggesting overall hepatocyte injury (Messner et al., 2019). Other proposed mechanisms of toxicity include ROS generation, endoplasmic reticulum stress, alterations to bile canaliculi dynamics and destruction to lipid membranes (Perez and Briz, 2009; Sharanek et al., 2016). In physiological conditions, BAs aid in the digestion of lipids by emulsifying hydrophobic compounds into micelles for easy digestion and excretion (Hofmann, 1999b; Chiang, 2013). However, when present in excess levels, BAs bind to lipid components within the cellular membrane affecting stability, fluidity and permeability of the membrane, which can lead to cell death (Hofmann, 1999a; Zhou et al., 2009; Maldonado-Valderrama et al., 2011).

## 4 Conclusion

DIC represents the most clinical manifestation of DILI, with BAs being identified as the causative agents of toxicity. Research in rat hepatocytes postulated BA mediated mitochondrial dysfunction however there are limitations with these studies. It is important to note that there are vast differences in BA concentrations and compositions in rodents and humans, with rats containing high levels of the less-toxic, hydrophilic bile salt taurocholic acid, whereas humans produce larger quantities of the more-toxic glycine-conjugated BAs (Woolbright and Jaeschke, 2015). Due to this, rodent hepatocytes in vitro are more sensitive to glycine-conjugated BAs as they are never exposed to these bile salts under in vivo conditions leading to conclusions that are not accurate reflections of the pathophysiology in humans (Marrero et al., 1994; Woolbright and Jaeschke, 2015). Furthermore, research in HepG2 cells has postulated that BAs induce mitochondrial dysfunction due to a decrease in MMP and induction of the mitochondrial apoptotic pathway (Rolo et al., 2004). Whilst these findings are not disputed, the research in this manuscript offers greater clarifications into the mechanisms of toxicity of DIC, revealing that mitochondrial toxicity does not precede cytotoxicity. Evidence from this research suggests that other mechanisms of toxicity, are responsible for the cytotoxic potential of BAs and thus induce the activation of the mitochondrial apoptotic pathway but as a downstream event of toxicity. The time-dependent alterations in MRP2 function and expression were consistent with the stages at which cytotoxicity occurred, however are not attributed to causing the cell death in HepaRG cells. Changes in MRP2 dynamics could have initiated other proposed mechanisms of BA-induced toxicity such as destruction of the lipid membrane, ROS generation, bile canaliculi alterations or endoplasmic reticulum stress, but not by targeting the mitochondria (Perez and Briz, 2009).

Isolated mitochondria are extensively used in mechanistic studies and to determine direct interactions between a compound and the mitochondria. Whilst the use of isolated mitochondria is valuable in determining direct mechanisms of toxicity, their lack of cellular context means their physiological relevance is limited (Brand and Nicholls, 2011). To date, experiments in isolated mitochondria have exposed BA-mediated mitochondrial toxicity as a potential mechanism of toxicity in DIC. However, the translatability of these effects within an appropriate cell model have not been completed. HepaRG cells are a suitable model for DIC studies due to their functioning biliary transporters, ability to be chronically dosed and similar levels of phase 1–3 enzymes as PHH. To the best of our knowledge, this is the first study focussing on the mitotoxicity of BA mixtures in isolated mitochondria and whole cells simultaneously. Overall, it was demonstrated that mitochondrial toxicity does not precede cytotoxicity in HepaRG cells following short and long-term exposure to BA mixtures. The generation of mitochondrial toxicity seen in isolated mitochondria did not translate to the whole cell model, even when key transporters were inhibited to manipulate static levels of BAs. Overall, this research has demonstrated that there are important mechanistic differences when BAs interact at the organelle level versus the whole cell. This has important implications when considering the role of BA accumulation in DILI. These findings are not only relevant to DIC but to all toxicological testing in which mitochondrial dysfunction has been detected and raises important questions regarding limitations of the use of isolated mitochondria.

## Funding

This work was supported by the Centre for Drug Safety Science supported by the Medical Research Council, United Kingdom (grant number G0700654); and SP was funded by MRC and Biologie Servier through an MRC-CASE studentship.

## Declaration of Competing Interests

The authors have no conflict of interests to disclose

## Appendix A Supplementary data

Supplementary figures Image 1
