# Supplementary material for: Differential toxic effects of bile acid mixtures in isolated mitochondria and physiologically relevant HepaRG cells
Source: Toxicol In Vitro. 2019 Dec;61:104595. doi: 10.1016/j.tiv.2019.104595 (PMC6853172; doi:10.1016/j.tiv.2019.104595)
Supplement: Supplementary file 1 — Supplementary figures [file mmc1.docx]

**Supplementary figures**

**
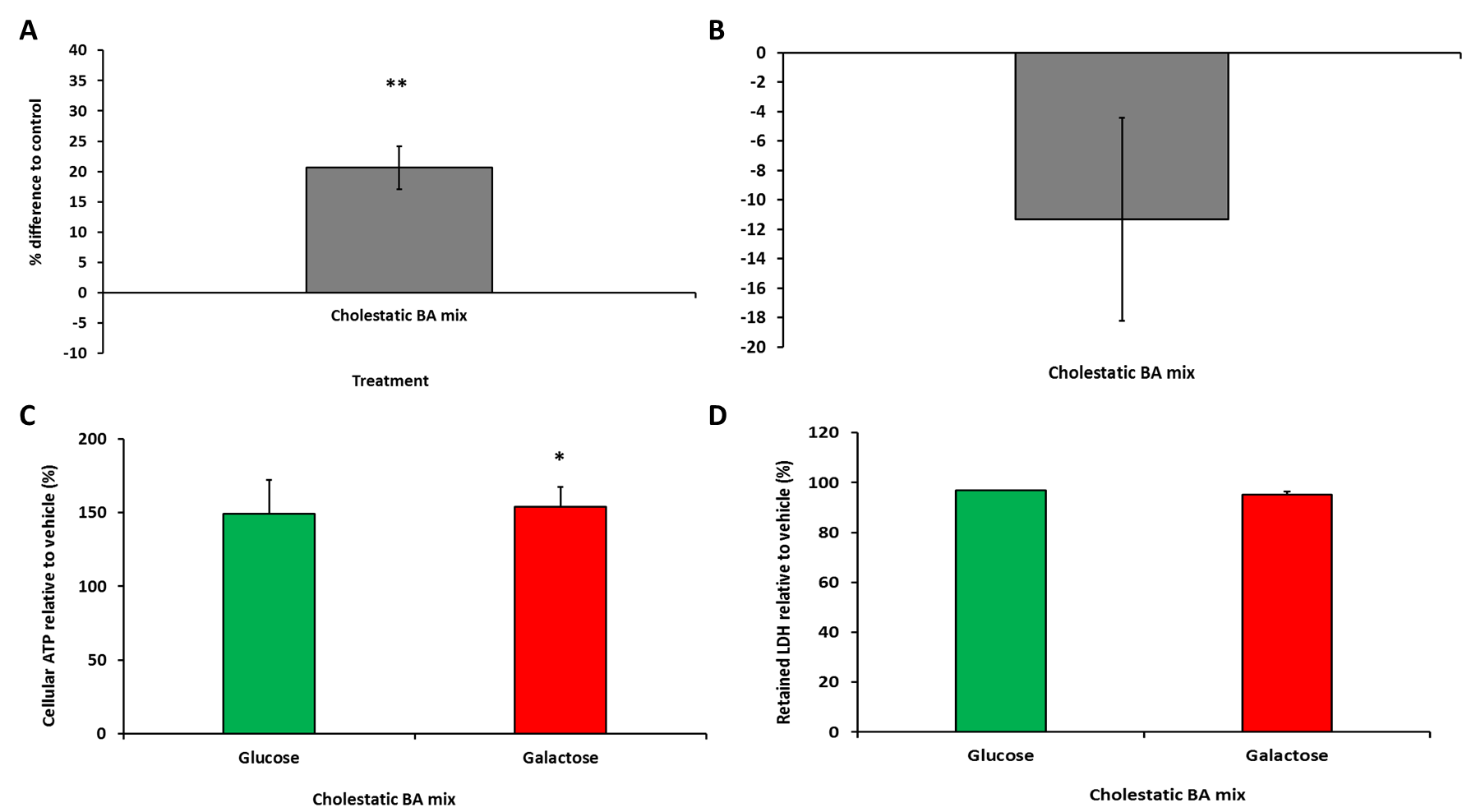
**

**Supplementary Figure 1: The mitochondrial effects of the additional bile acid (BA) mixture with a composition based upon the concentrations of BAs detected within the bile of patients with cholestasis in isolated mitochondria and HepaRG cells.** (A) % difference in MMP and (B) optical density at kinetic read 20 for isolated mitochondria acutely treated with the cholestatic BA mixture compared to control. (C) Changes in cellular ATP in glucose and galactose media and (D) retained LDH in glucose and galactose media in HepaRG cells after 24 hours treatment with the cholestatic BA mixture compared to vehicle control. ATP values have been normalised to µg protein per well. There were no significant differences between ATP levels between glucose or galactose media indicating an absence of mitochondrial toxicity in the HepaRG cells. Statistical significance compared with control; * P < 0.05, ** P < 0.01, *** P < 0.001, ****P < 0.0001. Data are presented as ± SEM of n = 3 experiments.


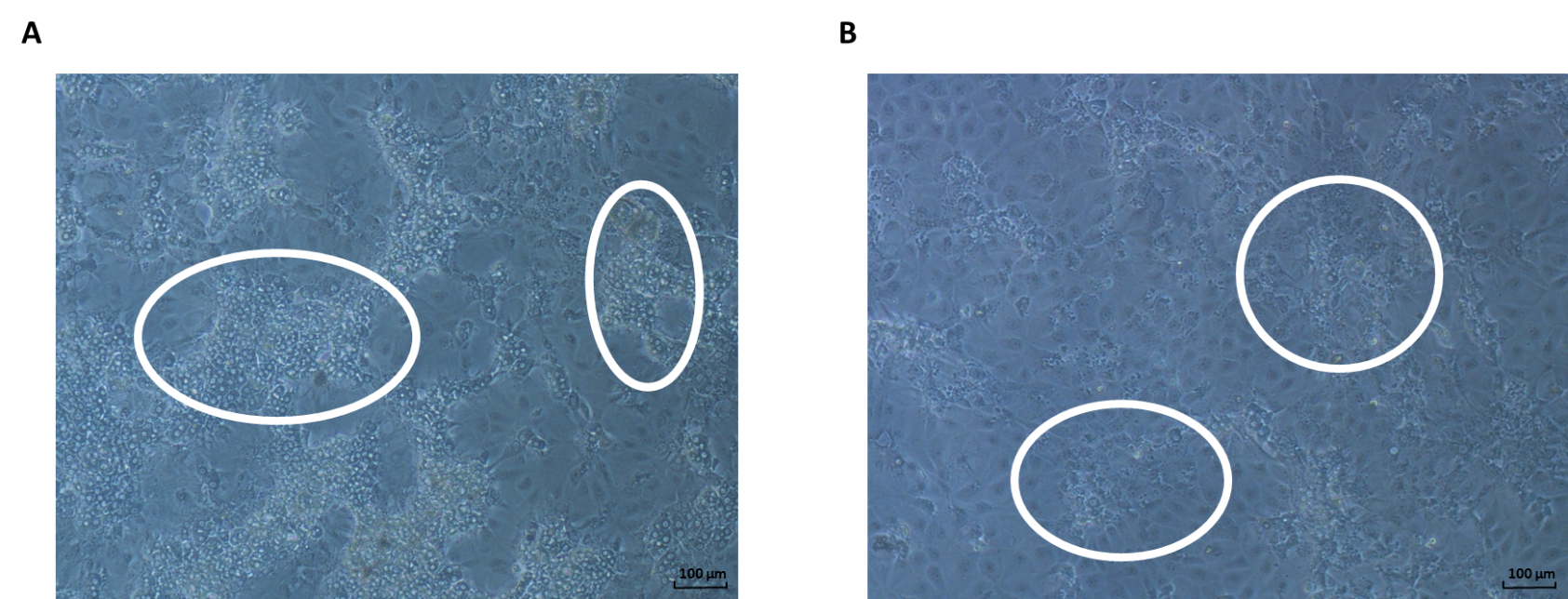


**Supplementary Figure 2: Changes in HepaRG cell morphology following 2 weeks treatment with the 1000 x BA mix.** HepaRG cells are a heterogeneous population containing both hepatocytes and primitive biliary-like cells. Circled areas represent examples of the hepatocyte clusters. (A) Morphology of vehicle treated HepaRG cells. (B) Morphology of HepaRG cells following 2 weeks 1000 x BA mix treatment. There is a loss of the hepatocyte clusters following BA mix treatment. Images were taken using a Nikon Eclipse TS100 optical microscope using a 10 x objective. Scale bar = 100 µm.
